# Altered molecular repertoire of immune system by renal dysfunction in the elderly: is prediction and targeted prevention in the horizon?

**DOI:** 10.1186/1878-5085-4-17

**Published:** 2013-06-21

**Authors:** Cheng-Lin Lang, Min-Hui Wang, Kuan-Yu Hung, Chih-Kang Chiang, Kuo-Cheng Lu

**Affiliations:** 1 Division of Nephrology, Department of Internal Medicine, Cardinal Tien Hospital, Yong-He Branch, Taipei 234, Taiwan; 2 Division of Nephrology, Department of Internal Medicine, Cardinal Tien Hospital & School of Medicine, Fu-Jen Catholic University, New Taipei City 231, Taiwan; 3 Division of Nephrology, Department of Internal Medicine, National Taiwan University Hospital & College of Medicine, National Taiwan University, Taipei 10048, Taiwan

**Keywords:** T cell differentiation, Age, Hemodialysis, Vitamin D, Immunosenescence, Integrative medical approach, Target prevention

## Abstract

**Background:**

Patients on chronic hemodialysis (HD) have impaired cellular and humoral immunity. The percentage of elderly people among the total population in Taiwan is increasing dramatically, and HD is the primary alternative for renal replacement therapy when renal function declines. Activated vitamin D is widely used in HD patients with secondary hyperparathyroidism (SHPT) and is a well-known immunomodulatory agent. Personalized medicine and integrative medical approach has been a trend in current clinical practice. Can we improve their immune function using vitamin D in spite of the mineral aspect? Here, we investigated the relationship between serum 25-hydroxyvitamin D (25(OH)D) level and T cell differentiation in chronic HD patients.

**Methods:**

Forty patients with chronic HD were enrolled. HD patients with SHPT had been treated with activated vitamin D for 3 months. Peripheral blood mononuclear cells obtained from the patients were cultured and stimulated by mitogens, and T cells were analyzed by flow cytometry. Serum 25(OH)D levels were detected by enzyme-linked immunosorbent assay.

**Results:**

The incidence of T cell differentiation to the T helper cell (Th)2 subtype was more prevalent in the elderly group than in the controls (*p* = 0.001). Th2 differentiation was also correlated with age (*p* = 0.004) and serum 25(OH)D levels (*p* < 0.05). After treated with activated vitamin D, the level of Th1 cytokines decreased while the Th2 cytokine level increased in the sera (*p* < 0.05). The T cell differentiation tended toward the Th2 subtype (*p* = 0.027) after treatment of activated vitamin D in SHPT patients.

**Conclusions:**

These results demonstrated that Th2 differentiation is correlated with age and the serum 25(OH)D level of patients. Treatment with activated vitamin D influenced T cell differentiation and cytokine expression in SHPT patients. Taking vitamin D is the possible prediction and targeted treatment in the immune dysfunction in chronic HD patients.

## Overview

The proportion of elderly people is increasing dramatically in Taiwan, and hemodialysis (HD) is the primary alternative for renal replacement therapy in cases of renal function decline. However, the immune system of end-stage renal disease (ESRD) patients is impaired. In these patients, monocytes are preactivated and they overproduce cytokines such as tumor necrosis factor-alpha (TNF-α), interleukin (IL)-1, IL-6 and IL-10 [1,2]. Chronic kidney disease (CKD) patients have lower percentages of peripheral T lymphocytes, both CD4^+^ and CD8^+^, and B lymphocytes in the blood [3]. Further, soluble B lymphocyte markers increase in CKD patients [4], but studies have also shown an increased incidence of apoptosis in B cells [5]. Because of their impaired immunity, these patients are more susceptible to infection, have a higher risk of malignancy and demonstrate a poor response to vaccination [6-8]. Many studies have shown that both naive and acquired immune systems are impaired in HD patients. The HD procedure itself damages the skin barrier and leaves patients vulnerable to skin infections. HD patients, especially those who are elderly, are usually too malnourished to produce adequate immune responses. Heart failure and malnutrition/inflammation are prognostic factors related to mortality in these groups [9].

Calcitriol, or 1,25-dihydroxyvitamin D_3_ (1,25(OH)_2_D_3_), is a well-known endocrine regulator of calcium homeostasis. Calcitriol receptors are present on many immune cells, and therefore, this compound has an immunomodulating effect. 1,25(OH)_2_D_3_ promotes monocyte-to-macrophage differentiation and diminishes the production of proinflammatory cytokines and chemokines by macrophages. Further, 1,25(OH)_2_D_3_ inhibits the proliferation, maturation and differentiation of dendritic cells [10]. Vitamin D has direct and indirect effects on T and B cells and modifies their response to activation. It plays an important role in adaptive immune responses. 1,25(OH)_2_D_3_ predominantly decreases the T helper cell (Th)1 and Th17 subtypes, which are involved in cell-mediated immunity, and facilitates the development of the Th2 and Treg subtypes, which are involved in the humoral immune response and have anti-inflammatory and anti-atherogenic properties [11,12]. The effects of 1,25(OH)_2_D_3_ on B cells include inhibition of B cell proliferation, differentiation to plasma cells and production of immunoglobulins [13]. Vitamin D supplement may have the impact on the risk and/or the course of multiple sclerosis [14]. Therefore, 1,25(OH)_2_D_3_ exerts multiple immunomodulatory effects via the innate and adaptive immune pathways. Calcitriol is widely used for treating secondary hyperparathyroidism (SHPT) in chronic HD patients, and the immunomodulating effects of calcitriol in HD patients with SHPT have been reported [12,15].

A body of evidence suggests that innate immunity does not get impaired and is upregulated with healthy aging [16]. However, changes in T cell subpopulations are seen most often: naive T cells decrease, whereas memory T cells increase. Published data support that human aging is associated with a decline in Th1 immunity and enhancement of Th2 immunity [17,18]. Therefore, how would their immunity change while we use the vitamin D? Can we improve their immune function using vitamin D in spite of the mineral aspect? In this study, we investigated the effect of age and vitamin D levels on T cell differentiation and cytokine expression in chronic HD patients and the effects T cell differentiation and cytokine expression after treatment with activated vitamin D in SHPT patients.

## Methods

### Patients

Twenty elderly patients (aged ≥65 years) who had regular HD for at least 3 months were enrolled. Twenty chronic HD patients aged <65 years comprised the control group. Only patients who did not take vitamin D or vitamin D analogues in the past 3 months were included. Exclusion criteria comprised systemic infection or malignancy, use of immunosuppressive medication, HIV infection and untreated autoimmune disease. Dialysis was performed with bicarbonate dialysate and a high-flux polysulfone membrane dialyzer (Kawasumi Laboratory Inc., Tokyo, Japan) without reprocessing.

### Biochemical analysis

Midweek predialysis biochemical and hematological parameters were obtained. Hemoglobin and hematocrit were assessed using the XT100i automated chemistry analyzer (Sysmex, Kobe, Japan). Prehemodialysis blood urea nitrogen, creatinine, total calcium, serum phosphate and serum albumin were determined using the LX20 automated analyzer (Beckman Coulter, Fullerton, CA, USA). The intact parathyroid hormone level in serum was determined using the ADIVIA Centaur analyzer (Siemens, Washington, DC, USA).

### Isolation and culture conditions of peripheral blood mononuclear cells

Blood samples (10 mL) were collected just before the midweek HD session. Peripheral blood mononuclear cells (PBMCs) were isolated from the buffy coats with Ficoll-Paque (Pharmacia Biotec AB, Uppsala, Sweden) density gradient centrifugation. Next, cell numbers were determined, and the cells were cultured at 2 × 10^6^ cells/mL in RPMI-1640 (Gibco BRL, Paisley, Scotland) medium supplemented with 10% fetal calf serum (Biochrome KG, Berlin, Germany) and antibiotics (100 IU/mL penicillin, 100 μg/mL streptomycin and 0.25 μg/mL amphotericin B). PBMCs were primary stimulated with 2 μg/mL phytohemagglutinin-L (PHA-L, Roche, Mannheim, Germany) for 48 h. The PBMCs were secondarily stimulated for 4 h with 20 ng/mL phorbol myristate acetate (PMA, Sigma, St. Louis, MO, USA) and 1 μM/mL ionomycin (Sigma) in the presence of the intracellular cytokine transport inhibitor GolgiStop (1 μL/mL, BD Biosciences, San Jose, CA, USA). The cells were cultured in a humidified incubator at 37°C with 5% CO_2_. Cells were then stained for surface and intracellular cytokine markers.

### Cytokine measurements

The amounts of IL-2, IL-4, IL-5 and interferon-γ (IFN-γ) in the serum were determined by enzyme-linked immunosorbent assay (ELISA), according to the manufacturer’s instructions (eBioscience, San Diego, CA, USA). All samples were analyzed in triplicate.

### Antibodies

All antibodies were fluorescence-labelled. Phycoerythrin-Cy5 (PE-Cy5)-labelled anti-CD4 (clone RPA-T4) was used to detect the surface antigen of T cells. Fluorescein isothiocyanate (FITC)-labelled anti-IL-4 (clone MP4-25D2) and phycoerythrin (PE)-labelled anti-IFN-γ (clone 4S.B3) were used for intracellular staining. All of the antibodies were obtained from eBioscience (San Diego, CA, USA).

### Intracellular cytokine staining

After stimulation by PHA and PMA/ionomycin, aliquots of 10^5^ cells/tube were used for intracellular cytokine staining. The surface cytokine was stained with PE-Cy5-labelled anti-CD4 antibody. After permeabilization, cells were double-stained with anti-cytokine antibodies (FITC-labelled anti-IL-4 and PE-labelled anti-IFN-γ). Cells were analyzed within 24 h using a FACSCalibur (Becton Dickinson, Franklin Lakes, NJ, USA) with CellQuest software (Becton Dickinson). Data were expressed as the percentage of double-stained positive cells (anti-CD4 and anti-cytokines).

### 25(OH)D measurement

The amount of 25(OH)D in serum was determined by ELISA according to the manufacturer’s instructions (Immundiagnostik AG, Bensheim, Germany). The serum samples were measured in triplicate.

### Treatment group

Activated vitamin D (Calcijex®, Abbott Laboratories, IL, USA) was prescribed for patients with secondary hyperparathyroidism for 3 months. The dosage was prescribed according to the Kidney Disease Outcomes Quality Initiative (NKF KDOQI) guidelines. Treatment was withheld in cases of severe hypercalcemia (serum total calcium >10.5 mg/dL), or hyperphosphatemia (serum phosphorus level >7 mg/dL), or where Ca × P product was greater than or equal to 70 mg^2^/dL^2^. Analysis of T cell differentiation and cytokine expression would be repeated 3 months after the start of treatment.

### Statistical analysis

Continuous variables were expressed as means ± standard deviation, and categorical values were expressed in percentages. The differences in elderly and control groups were analyzed by independent *t* tests. Pearson correlations were derived to evaluate the possible correlations between biological markers and continuous variables. Significance was defined as *p* < 0.05. Statistical analyses were performed with the Statistical Package for the Social Sciences, version 17.0 (SPSS Inc., Chicago, IL, USA).

### Ethics

All patients gave informed consent for this study, and the study was reviewed and approved by the Human Ethics Committee of the Cardinal Tien Hospital, Taiwan (CTH-97-2-5-057).

## Results

### General characteristics of the study subjects

The clinical characteristics of study subjects are shown in Table 1. HD patients of different ages had no significant differences in their baseline data, such as causes of ESRD, diabetes and hepatitis condition and dialysis vintage.

**Table 1:** Clinical characteristic of subjects (mean ± SD) in the different age groups

	**Elder group**	**Control group**
	**(≧65 y/o)**	**(<65 y/o)**
Number of patients	20	20
Age (y/o)	71.9 ± 4.5	51.4 ± 10.5*
Sex (M/F)	6/14	10/10
DM/non DM	14/6	13/7
HBsAg (+/−)	2/18	4/16
Anti-HCV (+/−)	3/17	2/18
Cause of end-stage renal disease	DM: 14; HTN :3	DM: 13; HTN :2
	CGN: 1; Obstructive: 2	CGN: 5
Dialysis vintage (year)	6.1 ± 4.7	5.3 ± 3.7

^*^p < 0.05 versus elder group.

The HD patients of different ages had no significant differences in their biochemical and haematological exams. The data showed that only albumin was lower in the elderly group (3.93 ± 0.30 versus 4.19 ± 0.20, *p* = 0.03, Table 2).

**Table 2:** Biochemistry and flow cytometry results of subjects (mean ± SD) in the different age groups

	**Elder group**	**Control group**
	**(≧65 y/o)**	**(<65 y/o)**
Hemoglobin (g/dL)	10.6 ± 1.7	9.9 ± 1.2
Hematocrit (%)	32.4 ± 5.3	30.2 ± 3.9
Albumin (g/dL)	3.9 ± 0.3	4.2 ± 0.2*
Predialysis BUN (mg/dL)	59.9 ± 13.0	68.3 ± 17.1
Predialysis Cre (mg/dL)	9.4 ± 1.8	10.7 ± 2.6
Kt/V	1.6 ± 0.3	1.6 ± 0.3
Total calcium (mg/dL)	9.3 ± 0.8	9.5 ± 0.9
Phosphorus (mg/dL)	4.8 ± 1.2	5.8 ± 1.9
Intact PTH (pg/dL)	236.8 ± 241.0	389.7 ± 435.5
25(OH)D (pg/dL)	21.2 ± 10.2	19.6 ± 9.9
Th1 (%)	3.4 ± 8.9	10.86 ± 12.8*
Th2(%)	56.7 ± 32.9	23.9 ± 22.7**
Serum IL-2 (pg/ mL)	17.63 ± 16.2	17.2 ± 10.3
Serum IFN-γ (pg/ mL)	51.2 ± 27.2	50.5 ± 37.2
Serum IL-4 (pg/ mL)	0.75 ± 1.4	0.18 ± 0.4
Serum IL-5 (pg/ mL)	68.3 ± 44.6	61.7 ± 36.0

^*^p < 0.05 versus elder group; ^**^p < 0.001 versus elder group.

### T cell differentiation correlated with serum 25(OH)D levels

**Figure 1:**
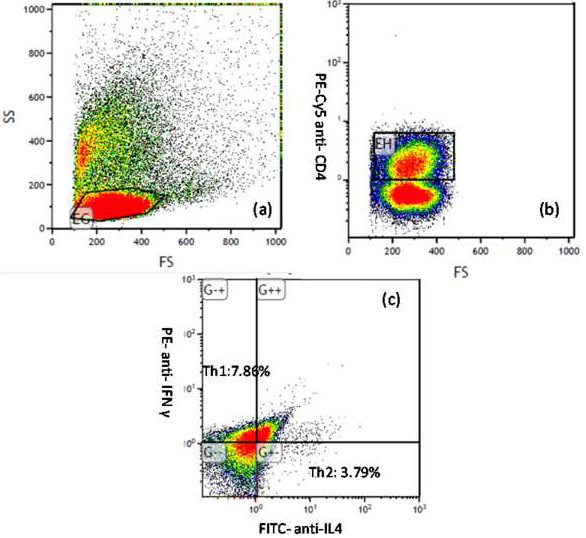
**PBMCs from patients after culture with stimulation mitogen were then triple-stained for surface and intracellular cytokine antibodies.** A total 10,000 events were acquired. **(a)** Lymphocyte selection, **(b)** chose the PE-Cy5-labelled positive cells as Th cell, **(c)** results were expressed as percentage of cells stained in total events.

To evaluate the T cell response to mitogens, we analyzed the frequency of cytokine-producing T cells within the PBMCs of our patients. A total of 10,000 events were acquired and analyzed after lymphocyte selection. We collected the PE-Cy5-labelled anti-CD4^+^ cells as Th cells, with Th1 cells defined as PE-labelled anti-IFN-γ positive and Th2 cells as FITC-labelled anti-IL-4 positive (Figure 1).

**Figure 2:**
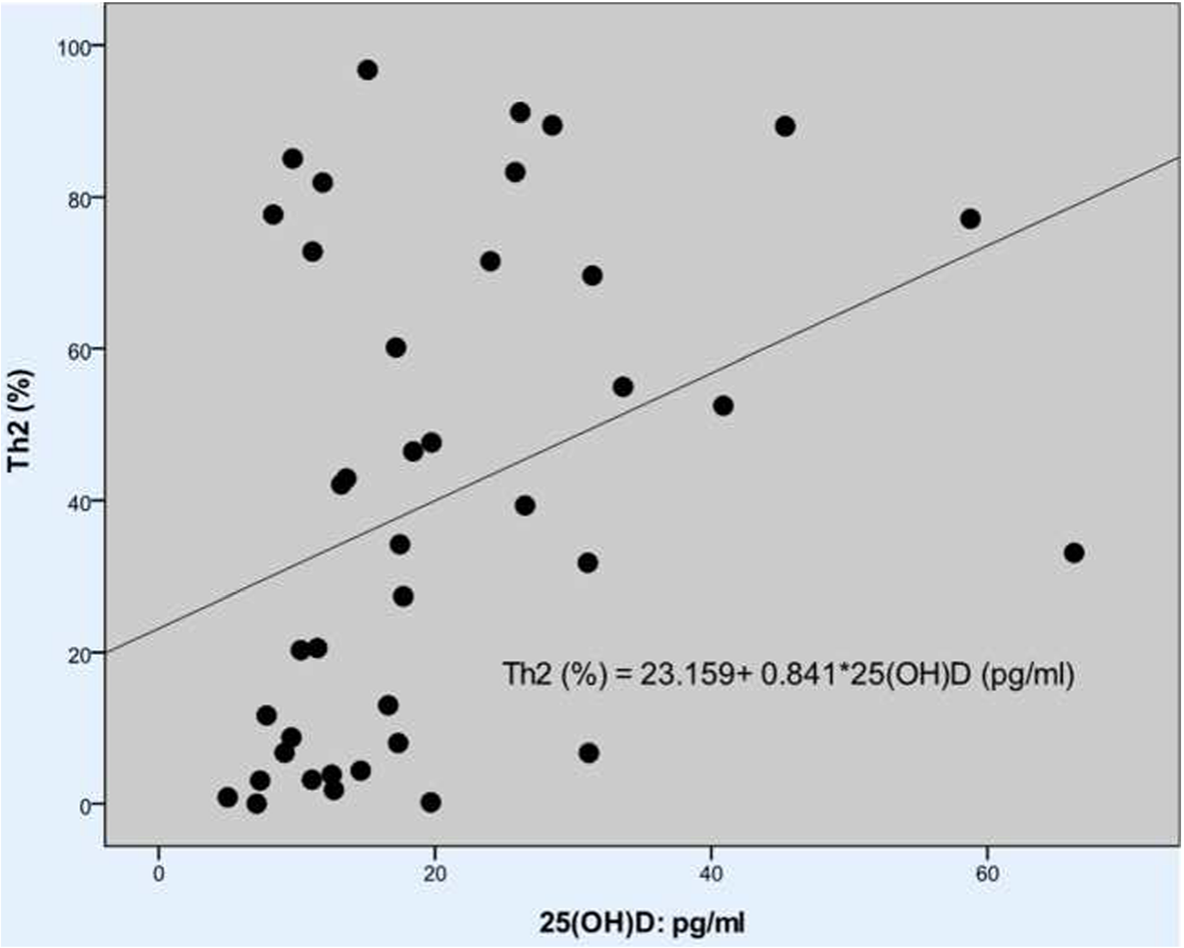
T cell differentiation correlated with serum 25(OH)D level.

Regardless of age, Th2 differentiation was more prevalent than Th1 in our patients (40.29% ± 32.5% versus 7.14% ± 11.5%). Further analysis disclosed that the percentage of Th1 and Th2 differentiation was linked to serum 25(OH)D levels (*r* = −0.319, *p* < 0.05 and *r* = 0.354, *p* < 0.05, respectively). The higher the serum 25(OH)D levels, the greater was the Th2 cell differentiation in PBMCs (Figure 2).

### T cell differentiation to the Th2 subtype was more prevalent in the elderly group

**Figure 3:**
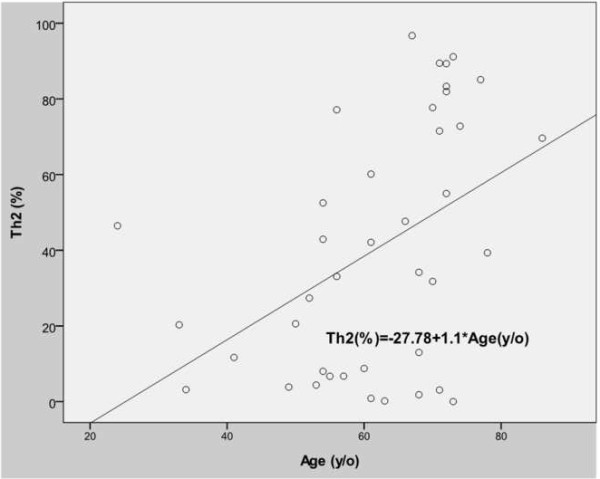
T cell differentiation correlated with the age.

The incidence of T cell differentiation to the Th2 subtype was more prevalent in the elderly group (56.73 ± 32.90 versus 23.85 ± 22.73, *p* = 0.001). Th2 cell differentiation was also correlated with age (*p* = 0.004). The proportion of T cell differentiation to the Th2 subtype increased with age (Figure 3).

### Treatment with activated vitamin D for 3 months

Nineteen patients had serum iPTH levels above 300 pg/ dL. During the course of the study, three patients dropped out due to personal reasons, while six patients had refractory hypercalcemia or hyperphosphatemia. The remaining ten patients were treated with activated vitamin D for 3 months. At the end of that time, they showed no significant changes in the serum 25(OH)D level and iPTH level (Table 3).

**Table 3:** T cell cytokines, T cell differentiation and serum iPTH and 25(OH)D changes in ten patients treated with activated vitamin D

	**Tx with Calcijex® according to serum iPTH**	
	**Before treatment**	**After treatment**
Number of patients	10	10
iPTH (pg/ml)	712.1 ± 237.1	509.1 ± 337.6
25(OH)D (ng/ml)	19.6 ± 14.4	21.3 ± 7.9
Th1 (%)	6.2 ± 4.9	5.6 ± 4.4
Serum IL-2 (pg/ml)	19.6 ± 9.8	16.7 ± 8.3**
Serum IFN-γ (pg/ml)	39.1 ± 13.3	37.4 ± 12.7*
Th2 (%)	40.2 ± 20.9	44.9 ± 22.8*
Serum IL-4 (pg/ml)	0.3 ± 0.3	1.6 ± 0.7**
Serum IL-5 (pg/ml)	76.4 ± 10.4	95.1 ± 8.7**

^*^p < 0.05 versus elder group; ^**^p < 0.001 versus elder group.

**Figure 4:**
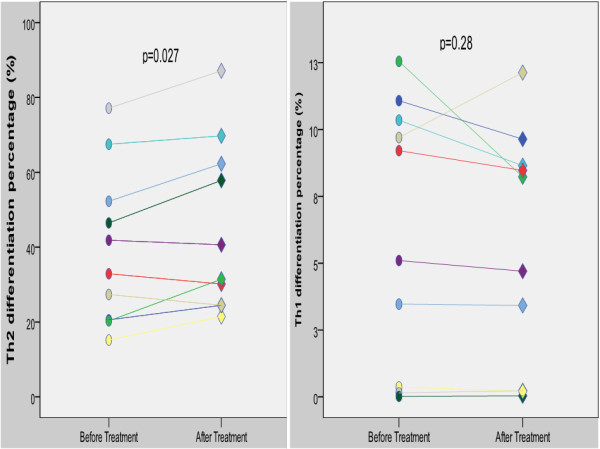
T cell differentiation change before and after treatment with activated vitamin D in the hemodialysis patient with secondary hyperparathyroidism.

In the SHPT patients, a significant difference in cytokine level was found before and after treatment. In summary, Th2 cytokine expression (IL-4 and IL-5) had increased, while Th1 cytokine (IL-2 and IFN-γ) expression had decreased after treatment. In addition, Th2 cell differentiation had increased after treatment with activated vitamin D (Figure 4).

## Conclusion

In conclusion, Th cell differentiation correlates with age and serum hydroxyvitamin D levels in patients on chronic maintenance HD. Immunosenescence is also observed in HD patients with alterations in T cell differentiation, cytokine dysregulation and preactivated antigen-presenting cells. Modification of the immunosenescence status in elderly patients on chronic HD is still a challenge. Supplementary treatment with vitamin D for chronic HD patients may not only treat secondary hyperparathyroidism but may also reduce inflammation, by increasing the levels of Th2 cytokines and Th2 cell differentiation. Taking vitamin D is the treatment tailored to the HD patients and the main prevention in the impaired immune system. Further study is needed to clarify the effect of nutritional vitamin D supplementation on reduce cardiovascular risk or improve vaccination responses in HD patients.

## Expert recommendations

In the predictive, preventive and personalized medicine aspect, how to improve the immune dysfunction in the chronic HD patients remains the Achilles’ heel in clinical nephrology. Many studies showed lots of markers to alter their immunity, such as uremic toxin, oxidative stress, anemia, hyperparathyroidism and calcium and phosphorus metabolism. This study showed that T cell differentiation correlated with vitamin D levels and age in chronic HD patients. T cells differentiate into the Th2 subtype in patients with higher levels of vitamin D. The incidence of T cell differentiation to the Th2 subtype was more prevalent in the elderly group. Our results also revealed that treating HD patients with activated vitamin D led to a decrease in inflammatory Th1 cytokines and an increase in anti-inflammatory Th2 cytokines. While we use the activated vitamin D in our HD patients with SHPT, we also can improve their immunity function since Th2 cell has more anti-inflammatory and anti-atherosclerosis effects.

T cell functions are used as disease biomarkers of inflammation/sepsis and organ rejection and as biomarkers for monitoring the effects of immunosuppressive drugs [19]. Apart from the aging process, the presence of comorbidities such as atherosclerosis, cardiovascular disease and dialysis for ESRD lead to immune function deterioration in elderly persons. In the current study, T cell differentiation to the Th2 subtype was found to be correlated with age. The degree of Th2 differentiation increased with age. One possible explanation for these results is that healthy human aging is associated with a decline in Th1 and enhancement of Th2 responses [20-22]. In uraemia patients, the Th2 phenotype is also enhanced [23] and HD procedure contributes to the development of T cell lymphopenia by apoptosis induction [24]. Though, several authors independently observed that during HD, monocytes release inflammatory monokines, such as TNF-*α*, IL-1*β* and IL-6; these led to the theory that bio-incompatibility plays a major role in HD procedure [25]. In our study, we attenuate the bio-incompatibility effect difference with high-flux polysulfone membrane dialyzer in our patients. Another explanation is that Th1 cells easily undergo apoptosis in healthy elderly and HD patients. Further, there is a loss of telomerase activity and telomere length in the normal aging population [26]. In dialysis patients, Th1 lymphocytes had decreased expression of the anti-apoptotic molecule Bcl-2, which made the Th1 cells more susceptible to apoptosis [27]. Therefore, elderly chronic HD patients have increased Th2 differentiation after mitogen stimulation.

Another interesting finding is that Th2 differentiation was linked to serum 25(OH)D levels in our dialysis patients regardless of age, with higher levels of vitamin D correlating with more Th2 differentiation. Generally, Th2, not Th1, promotes anti-inflammatory and anti-atherogenic effects in the process. Several studies have shown that some complications in ESRD patients, including anaemia [28], lipid and insulin abnormalities, cardiovascular risks [29,30] and early mortality [31], would be improved after correction of vitamin D deficiency. However, long-term follow-up that adheres to the NKF KDOQI guidelines is needed of our dialysis patients after correction of vitamin D deficiency with supplemental vitamin D. From the perspective of public health, mass screening is one of the most practical and successful methods on identifying people with high, intermediate and low cardiovascular risks at one time [32]. From this viewpoint, supplement of activated vitamin D possibly improves their immunity function and reduce the cardiovascular risks in the HD patients.

Many studies show that dysregulation of cytokines is prevalent in normal elderly and dialysis patients. Dialysis patients produce a large amount of proinflammatory cytokines in T cells, including TNF-α, IL-1 and IL-6; they also have a higher expression of IL-2 in T lymphocytes [3]. In this study, we did not find a difference in the Th1 (IL-2, IFN-γ) and Th2 (IL-4 and IL-5) cytokines in the serum between the groups (Table 2). Healthy aging adults have higher levels of IL-6, IL-8, IL-10 and TNF-α, less IL-1 in their plasma or serum [16] and decreased IL-2 expression in T cells [17]. We suggest that HD elderly patients have no obvious different reaction in our cytokine study in the serum.

Age-related immunosenescence in the adaptive immune system, especially T cell apoptosis, has been extensively documented [33,34]. Immunosenescence in chronic HD patients includes loss of CD28 expression, skewed immune repertoire to the Th2 type, deficient T lymphocyte-dependent immune response and altered cytokine expression [34,35]. Cells of the adaptive immune system may be affected by nonspecific sequels of the aging process and HD, such as oxidative stress and glycation [12,18,35]. Imbalance between the production of free radicals and antioxidant defences of aging affect the immune system. The formation of advanced glycation end products (AGEs) can alter cell functions and cause a constant inappropriate cellular stimulation resulting in telomere shortening. The free radicals, AGEs and oxidative stress are common in our chronic HD patients and many studies to attenuate their effects in chronic HD patients now [36,37]. Therefore, HD patients are also ‘immunosenescence’ in their immune system. The deterioration of immune function in HD results from chronological age, not biological age. In elderly dialysis patients, Th2 differentiation is correlated with age and serum vitamin D levels. ESRD induces a clinical state of immunodeficiency associated with a higher incidence of infection and malignancy; therefore, it is challenging to improve immunity and restore immunosenescence in these patients.

In this study, we want to share our results to the healthcare that the Th2 cell differentiation is correlated with the serum 25(OH)D level and the age. We can use the activated vitamin D for our HD patients not only for the SHPT but also for their cellular immunity, possibly reduce inflammation, by increasing the levels of Th2 cytokines and Th2 cell differentiation.

## Competing interests

The authors declare that they have no competing interests.

## Authors’ contributions

All authors participated in the design of the study, performed the statistical analysis and drafted the manuscript. All authors read and approved the final manuscript.
